# Impact assessment of current knowledge gaps and mitigation strategies in clinical FLASH proton therapy through a systematic review

**DOI:** 10.3389/fonc.2025.1550264

**Published:** 2025-09-11

**Authors:** Anne H. zur Horst, Steven J. M. Habraken, Marta Rovituso, Yvonne L. B. Klaver, Kees H. Spruijt, Mischa S. Hoogeman

**Affiliations:** ^1^ HollandPTC, Delft, Netherlands; ^2^ Department of Radiotherapy, Erasmus MC Cancer Institute, University Medical Center Rotterdam, Rotterdam, Netherlands; ^3^ Department of Radiotherapy, Leiden University Medical Center, Leiden, Netherlands

**Keywords:** FLASH radiotherapy, UHDR, proton therapy, treatment planning, risk factors, review

## Abstract

**Introduction:**

Following first clinical trials, the development of FLASH proton therapy (FLASH-PT) into a mature treatment modality is ongoing, while physical and biological conditions underlying the FLASH effect remain uncertain. Our aim is to assess the impact of these uncertainties on clinical FLASH-PT through a novel approach.

**Methods:**

A systematic literature review was conducted to collect relevant *in vivo* preclinical studies as well as FLASH-PT treatment planning and delivery approaches. This information was used to perform an impact assessment: the FLASH-PT process from patient selection to treatment delivery was divided into steps, and seven FLASH conditions were defined. The FLASH conditions included physical, delivery-related, and radiobiological aspects. For each step and FLASH condition, scores were assigned based on the (i) criticality for clinical applications, (ii) current knowledge, and (iii) available mitigation strategies. These scores were combined to obtain an overall impact for all FLASH conditions ranging from insignificant impact not affecting clinical routine to severe impact causing severe complications for clinical translation.

**Results:**

In total, 14 preclinical and 27 treatment planning studies were identified. From these, 47 combined scores were reported in the impact assessment. A severe impact was found for patient selection in the context of radiobiological uncertainties for the robustness of the FLASH effect with respect to beam pauses and interruptions and for the evaluation of dose rate due to their importance in the treatment process combined with remaining unknowns. Moderate to insignificant impact was found for fractionation and FLASH-PT treatment delivery mode (transmission or Bragg peak beams), as these offer strategies to circumvent uncertainties. Overall, dose requirements, the use of multiple fields, and dose rate conditions emerged as the most crucial factors.

**Conclusions:**

Since uncertainties about the FLASH conditions hinder the utilization of its full pre-clinical potential in clinical practice, focusing future preclinical experiments to gain further phenomenological rather than only mechanistic insights on these aspects is recommended.

## 1 Introduction

Ultra-high-dose-rate (UHDR) irradiation has shown considerable potential to spare normal tissues while maintaining tumor control, referred to as the FLASH effect (1, 2). Various degrees of sparing have been observed in pre-clinical studies for different models, tissues, and endpoints (2, 3). The basic conditions for the FLASH effect have thus far been identified as average dose rates exceeding 40–100 Gy/s, with total irradiation times below 200–500 ms and total (fraction) doses of above 4–10 Gy (2, 4). Current evidence suggests that the phenomenon of the effect does not critically depend on sub-millisecond temporal fine structure of dose delivery, as it has been observed in experiments with particles with different temporal structures (5–9), e.g., electrons (10–15), photons (16), and protons (17–20).

In FLASH proton therapy (FLASH-PT), the highest overall dose rates are reached at the highest available energy of 230–250 MeV (21, 22), leading to transmission beams (TB) in foreseen neuro-oncological, head-and-neck, and thoracic applications. To combine the potential benefits of FLASH with the dose conformity in proton therapy (PT), Bragg peak (BP) FLASH-PT is under development. Conventional intensity-modulated PT (IMPT) relies on upstream energy modulation with a degrader—at the expense of scatter and loss of beam current—and the energy switching takes too long to be UHDR-compatible (21). Alternatives under development include static energy modulation devices, such as patient-specific 3D-printed ridge filters (23, 24).

While the biological and physiological mechanisms underlying the FLASH effect and its parameters are actively researched, the clinical development of UHDR is also advancing: treatment planning studies (25–29) and first patient treatments (30, 31) have already been conducted—for example, a recent clinical trial for FLASH-PT has demonstrated the technological feasibility in palliative single-fraction 8-Gy treatments of bone metastases with a single pencil beam scanning (PBS) transmission beam (31, 32). However, more advanced treatment planning and delivery approaches require a deeper understanding of the FLASH effect and its integration in a treatment workflow. This includes developments in quality assurance (33, 34), FLASH modeling (35), and assumptions about physics parameters, along with fulfilling clinical requirements for plan quality and technological delivery.

To date, a systematic approach to link knowledge about the FLASH effect and the development as a new clinical treatment modality for PT is missing. This can be approached by modifying frameworks such as the Failure Modes and Effects Analysis (FMEA) (36–39) to integrate insights from literature with current knowledge gaps and to evaluate their impact and risks in all steps of the treatment process.

The aim of this paper is to identify which aspects in biology and physics are essential for the development of FLASH-PT and identify unknowns as well as currently available mitigation strategies and solutions for these based on literature. In doing so, we aim to systematically and quantitatively assess the impact of different FLASH conditions on the treatment process.To that end, we performed a systematic literature review to extract *in vivo* pre-clinical data and treatment planning studies. We searched preclinical studies to quantify the physical FLASH conditions (dose, dose rate, fractionation, and beam pauses) and radiobiological aspects, and catalogued treatment planning approaches for FLASH-PT to gain insights on delivery-related FLASH conditions (delivery time, treatment mode). Based on the findings, our primary objective is to obtain an overall impact to identify comparatively low-risk first clinical applications, to guide clinical FLASH-PT treatment approaches, and to focus future pre-clinical research elucidating the FLASH effect.

## 2 Materials and methods

To assess the impact of remaining physical and radiobiological FLASH unknowns on the PT process, an impact analysis comprising several steps was developed: first, treatment process steps were defined, and knowledge gaps about the FLASH effect in that context were identified. Based on this, the focus for a systematic literature review on the FLASH effect was set, the results were categorized, and relevant conditions were defined.

The population included *in vivo* pre-clinical and treatment planning studies. UHDR irradiation (intervention) of above 40 Gy/s was compared to conventional dose rate irradiation (below 1 Gy/s). For the pre-clinical studies, outcomes were converted to FLASH enhancement ratios (FER) to quantify the FLASH effect. The FER is defined as the ratio of doses at ultra-high and conventional dose rates that are necessary to achieve the same biological effect. For the planning studies, approaches were catalogued and grouped in different categories. The data extraction and analysis characterized different FLASH conditions and informed the impact assessment.

The impact was assessed for each process step and FLASH condition by scoring (i) the process-related criticality, (ii) the available knowledge from literature, and (iii) the mitigation strategies and solutions from literature. A final impact for FLASH-PT was calculated from the scores.

### 2.1 Definition of treatment process steps and FLASH conditions for the impact assessment

**Figure 1:**
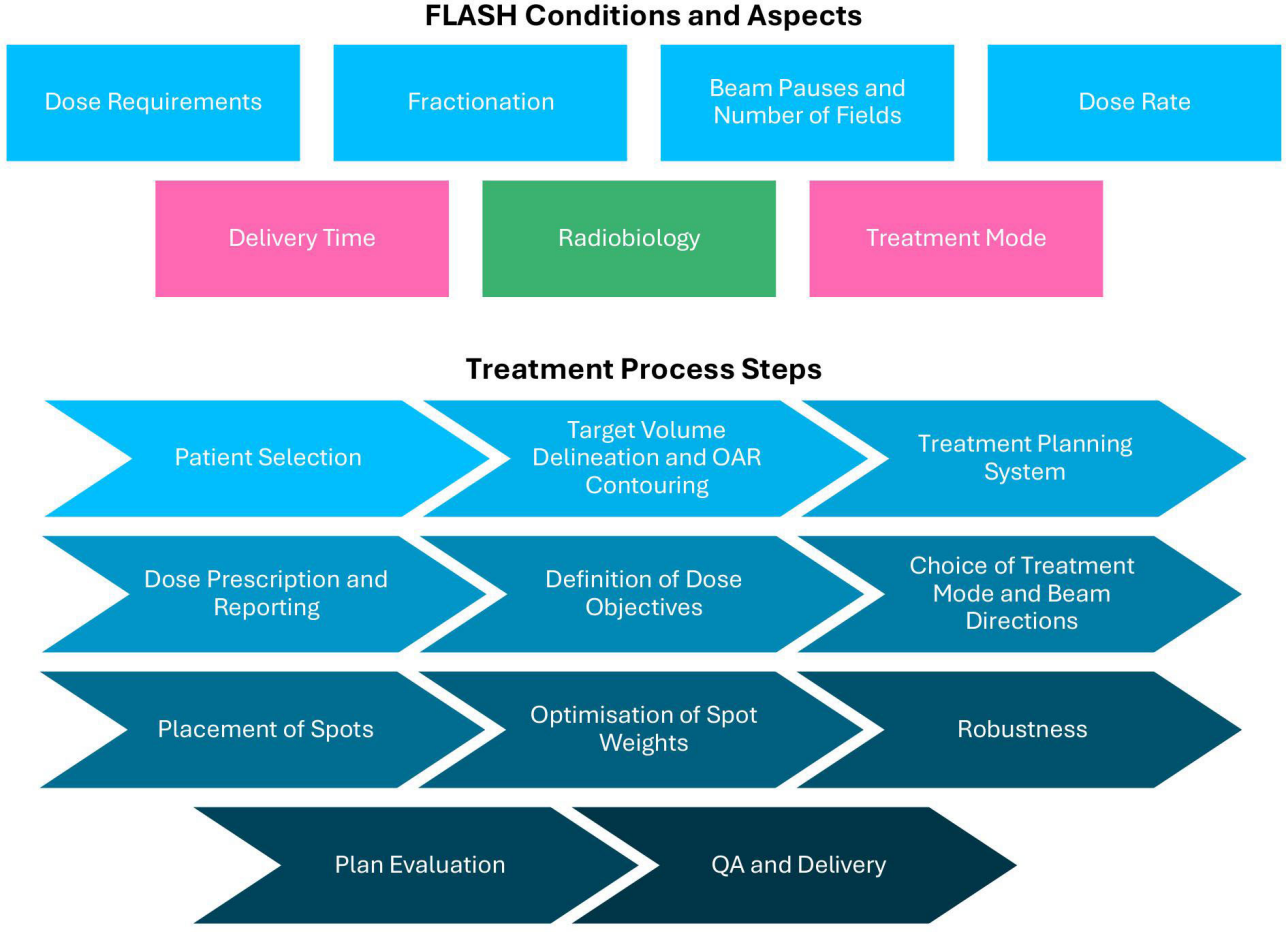
Overview of FLASH conditions (physical conditions: dose requirements, fractionation, beam pauses and number of fields, dose rate; delivery-related conditions: delivery time, treatment mode; tissue- und patient-related radiobiological aspects: radiobiology) and treatment process steps (from patient selection to QA and delivery).

The impact assessment was performed for clinical treatment planning and delivery of FLASH-PT with respect to seven conditions (see **Figure 1**). Physical FLASH conditions included dose, dose rate, fractionation, and beam pauses. Delivery time and treatment mode were defined as delivery-related aspects; additionally, the radiobiology (related to the variability of the biological response across tissues or patients) of FLASH was considered. The treatment process was divided into 11 steps: patient selection, target volume delineation and OAR contouring, treatment planning system (configuration), dose prescription and reporting, definition of dose objectives, choice of treatment mode (TB or BP) and beam directions, placement of spots, optimization of spot weights, plan robustness, plan evaluation, and QA and delivery.

### 2.2 Literature search

For the informed scoring and impact assessment, a systematic literature review was performed of the following:

- Published pre-clinical *in vivo* data that compares responses to UHDR and conventional (CONV) irradiations of normal tissue to characterize the physical FLASH conditions.
- Published treatment planning studies that provide insights into the generation, optimization, and evaluation of FLASH proton therapy treatment plans.

In a general search for FLASH radiotherapy and the FLASH effect, records published since 2014 were included (until March 8, 2024). Additionally, conference abstracts from the last 2 years were included. Details on the screened databases and search strategies for each of the databases can be found in **Supplementary Tables S1** and **S2** in the **Supplementary Material**. The review was not registered, a protocol was not prepared. The results were manually screened with Covidence (Covidence systematic review software, Veritas Health Innovation, Melbourne, Australia.; available at www.covidence.org) by one reviewer; duplicates and irrelevant entries were removed in the title and abstract screening. Records within the field of FLASH radiotherapy were then screened according to the exclusion criteria: comments, letters, editorials; micro-/mini-beam and other radiotherapy approaches, detectors and dosimetry, infrastructure, radiochemistry, mechanistic simulations, study protocols, reviews, and treatment planning with electrons were excluded. *In vivo* pre-clinical studies regardless of particle type and treatment planning studies for FLASH-PT were included in the full-text screening. Conference abstracts with a related published article were removed.

### 2.3 Data extraction from *in vivo* pre-clinical studies

*In vivo* pre-clinical records on normal tissue sparing with FLASH were assessed in full-text screening to quantify the physical FLASH conditions defined above. For the data extraction, CONV and UHDR data—irrespective of beam modality (electrons, protons, photons assumed to produce FLASH effect regardless of their fine structure (5–9))—needed to be available for the data extraction. CONV and UHDR were defined as averaged dose rates below 1 Gy/s and above 40 Gy/s, respectively. The dose rate is averaged over the entire treatment fraction. The extracted datasets included information about the biological system and irradiated volume, the endpoint and assessment time, beam characteristics, dose, averaged dose rate (ADR), other UHDR characteristics such as pulse width, dose per pulse and pulse frequency, and delivery time. While these experimental parameters may vary between beam modalities, we assumed that all experiments were descriptive of the FLASH effect based on the currently available information. Pre-clinical experiments will be referred to as pFLASH (proton FLASH) or eFLASH (electron FLASH) to specifically address pre-clinical work with the respective beam modality, in contrast to FLASH-PT which is used to address clinical considerations. As the aim was to characterize the physics parameters from the preclinical studies, negative results and tumor control studies (e.g (40, 41).,) were excluded for the description of these FLASH conditions; equivalent tumor control was assumed using UHDR. This may add to the publication and selection bias but aids in the characterization of the effect in terms of additional normal tissue sparing.

To quantify the FLASH effect for the parameters of interest, the FLASH enhancement ratio (FER) or a dose-modifying factor (DMF = 1/FER) was used; it depends on the biological system, endpoint and dose, and potentially on the physical FLASH condition. FER >1 indicates a normal tissue sparing through the FLASH effect. If FER was not directly provided in the extracted records, a conversion was performed depending on the data availability for each condition. If numerical experimental data was not available, it was digitized from graphs using WebPlotDigitizer (42). The data points themselves as well as their uncertainty (e.g., reported as standard deviations) were converted to the FER.

For the quantification of the FLASH effect as a function of dose, data from a recent review by Böhlen et al. (3) was extracted; its results were used for the characterization of dose with respect to FER. The fit function to describe the DMF with respect to dose was derived from normal tissue complication probability (NTCP) functions by Böhlen et al. (3), and its function parameters (dose threshold and maximal sparing effect) were calculated for individual and pooled data series. The inverted function (FER) was used for a graphical representation of their individual and pooled results in this work.

For the quantification of the FLASH effect as a function of dose rate, at least three different dose rates had to be reported in the datasets: a CONV and UHDR as previously defined, and intermediate dose rate(s). FER values needed to be extractable from the data; alternatively, ratios of intermediate dose rates between CONV and UHDR needed to be derivable. Similar to the approach by Böhlen et al. (3), the datapoints of the individual sets were then fitted and displayed with a logistic function (Equation 1, with dose rate and fit parameters, and FER^max^).


(1) $$F{\left(\dot{D}\right)}_{\dot{D_{50}},\;\gamma_{50}^{\text{FER}},\;{\text{FER}}^{\text{max}}}=\frac{{\text{FER}}^{\text{max}}}{1+e^{-\gamma_{50}^{\text{FER}}\left(\log(\dot{D)}-\dot{D_{50}}\right)}}+1$$

For the beam pause condition, pauses between subsequent pulses or fields had to be reported and FER values needed to be extractable from the data. For the fractionation condition, fractionation schemes (irradiations separated by at least 24 h) needed to be tested with CONV and UHDR. The results were interpreted based on the occurrence of the FLASH effect.

Due to the heterogeneity in the dose rate studies, little data on the beam pauses and the categorical representation of the fractionation condition, the individual results were not synthesized further. Nevertheless, general trends of these physical parameters may be derived from the results, even as the body of evidence will continue to grow with further pre-clinical studies.

The risk of bias was assessed using the SYRCLE Risk of Bias (RoB) (43) tool, which aligns with Cochrane but is tailored to animal studies. Ten domains were assessed in the categories (low/high/unclear) for the following types of bias: selection bias, performance bias, detection bias, attrition bias, reporting bias and other.

### 2.4 Data extraction from treatment planning studies

Treatment planning studies for FLASH-PT were assessed in full text screening to extract solutions for incorporating the FLASH effect in PT and mitigation strategies for knowledge gaps or necessary changes due to its incorporation. Records had to utilise at least one UHDR treatment mode (TB or BP) proposed for FLASH-PT and be tested on patient datasets. The extracted treatment planning studies included information about treatment mode, ridge filter design, treatment information (site, number of patient datasets, fractionation), number of fields, spot reduction methods, optimization technique, DR definition and evaluation methods. The studies were grouped according to their treatment mode, treatment sites, ridge filter design approaches, spot reduction approaches, dose rate metrics and optimization methods used.

The risk of bias was assessed using a modified RoB framework in the following categories: selection bias, data quality, study design, comparative measures, outcome measures, statistical analysis, reporting transparency, conflict of interest; see **Supplementary Table S8** in the **Supplementary Material**.

### 2.5 Impact assessment

The impact assessment for clinical FLASH-PT was performed considering deviations from the conventional approach. The method presented below will be referred to as “impact assessment”.

For the assessment of each FLASH condition in each treatment process step, it was first determined if there was any impact. If so, three sub-categories were scored on a 3-point scale [1 – 3]: the available knowledge, the criticality for clinical application, and proposed mitigation strategies and solutions proposed for FLASH-PT. A higher score indicates more critical, less understood, and less mitigable aspects, see **Table 1**. Finally, five impact levels were defined:

- Insignificant impact (scores 1, 2): Adjustments for planning and delivery are small, easily manageable and do not affect clinical routine.
- Minor impact (scores 3, 4): Updates to hardware and software require slight adjustments in protocols but are manageable with existing strategies.
- Moderate impact (scores 6, 8): Slight adjustments in planning and delivery that pose challenges in the process.
- Major impact (scores, 9, 12): Substantial modifications are required to mitigate for uncertainties and enable delivery effectively. More knowledge and solutions for planning and delivery is required.
- Severe impact (scores 18, 27): Planning and delivery is hindered by missing knowledge and mitigation strategies, causing severe complications for clinical translation.

**Table 1:** Scoring categories on a three-point scale used for the determination of final impact score.

Category	Score	Description
No		FLASH variable not relevant at this stage.
Low	1	**Criticality:** The FLASH variable imposes changes and limits but is not critical at this process stage. **Knowledge:** There is a reasonable (more than five) amount of independent literature for the FLASH variable for multiple clinical treatment regimes. **Mitigation:** The knowledge gap about the FLASH variable can be circumvented for several common clinical applications; mitigation strategies or solutions in treatment planning have been proposed and tested in multiple studies.
Intermediate	2	**Criticality:** The FLASH variable imposes changes and limits and is critical for some clinical applications at this process stage. **Knowledge:** There are some reports (less than three) of independent literature for the FLASH variable for limited clinical applications. **Mitigation:** The knowledge gap about the FLASH variable may be circumvented in some clinical applications; mitigation strategies or solutions in treatment planning have been proposed or only have a minor effect on the occurrence of the FLASH effect and the generation of clinically acceptable plans. Proposed solutions are under development and require further research.
High	3	**Criticality:** The FLASH variable imposes changes and limits and is critical for all clinical applications at this process stage. **Knowledge:** The effects of this FLASH variable are unknown. **Mitigation:** The knowledge gap about the FLASH variable cannot be circumvented and affects the occurrence of the FLASH effect or the generation of a clinically acceptable treatment plan. No solutions have been proposed.

Criticality for clinical applications, available knowledge, and proposed mitigation strategies and solutions were defined.

The scoring was performed independently by two of the authors active in the medical physics field with the information obtained through the literature review. Consensus was reached by those authors in a subsequent session; each sub-score was reviewed, and deviating scores were resolved. The scores of each sub-category were multiplied to a final impact score for each FLASH condition and treatment planning step and mapped to the impact levels defined above. The multiplication was chosen in order to put more weight on intermediate to high scores. For each parameter, the individual scores were summed.

For example, the effect of beam pauses on the choice of treatment mode and beam directions could be scored as highly critical (score: 3) for all clinical applications because beam interruptions may happen even for single-field treatments. Limited knowledge on beam pauses (score: 2) was available from the extracted data in the context of multiple fields in one fraction. While this uncertainty can be circumvented with single-field treatment, beam interruptions cannot, resulting in limited mitigation strategies (score: 2). After multiplication of these subscores, major impact was derived (total score: 12), indicating that more knowledge is needed.

## 3 Results

### 3.1 Literature search

**Figure 2:**
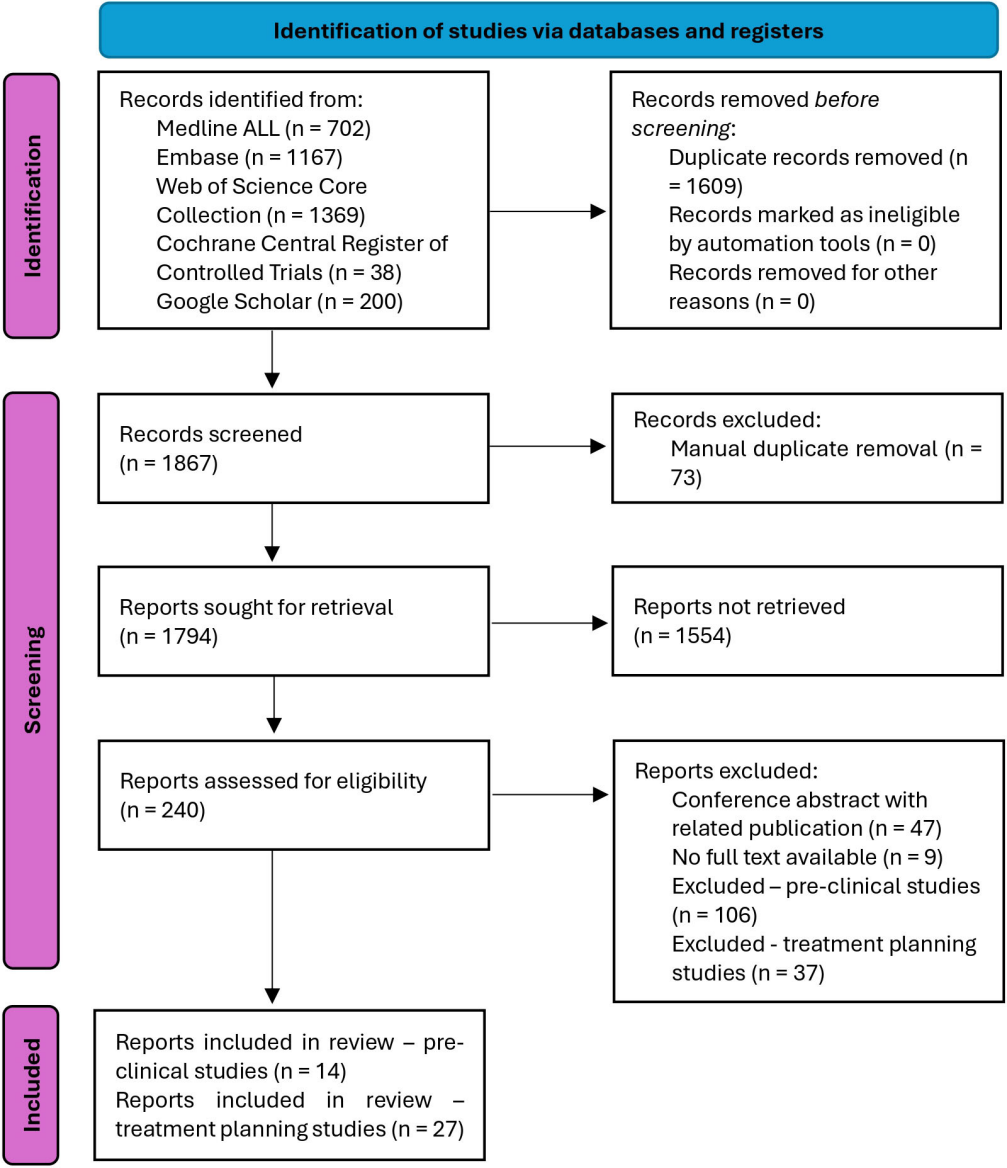
PRISMA flow diagram (44). In the identification step, the databases were searched, and duplicates were automatically removed. In the screening step, manual duplicates were removed, sought for retrieval according to the general exclusion criteria, and then assessed for eligibility according to the specific inclusion criteria for the pre-clinical and treatment planning studies. This led to 14 pre-clinical studies and 27 treatment planning studies having been included.

A total of 1867 records were found and screened. The screening strategy is summarized in a PRISMA flow diagram (44) in **Figure 2**. For the characterization of the physical FLASH conditions based on *in-vivo* pre-clinical studies, 14 records were included. Additionally, the screening yielded 27 treatment planning studies. Further details on the results from the screened databases and search strategies for each of the databases are provided in **Supplementary Tables S1**, **S2** in the **Supplementary Material**.

The risk of bias for both pre-clinical studies and treatment planning studies is provided in **Supplementary Tables S7**, **S8** in the **Supplementary Material**. For the former, the overall risk of bias in the individual studies was low or unclear. The baseline characteristics were adequately chosen, the reports did not suffer from reporting selective or incomplete outcomes. However, in some cases, it was unclear if the animals were randomly housed and the sequence was generated. More often, it was unclear if the allocation was concealed and if the investigators were blinded.

The risk of bias in the treatment planning studies was generally low as well. Concerns arose mainly from the selection of data due to the small number of patients used for testing. The data quality was generally good as clinical data had been used. The study design, comparative methods and outcome measures all raised low concerns. IMPT or VMAT were chosen for comparison with FLASH plans, which were designed according to the current state of knowledge, and conventional outcome measures of radiotherapy such as dose metrics and dose volume histograms were adapted for dose rate. Additionally, the reporting seemed transparent and complete. Since the tests were sometimes performed on a limited dataset, statistical analyses have not always been performed. Some studies received funding from industrial partners, which might pose as a conflict of interest.

#### 3.1.1 *In vivo* pre-clinical studies

**Figure 3:**
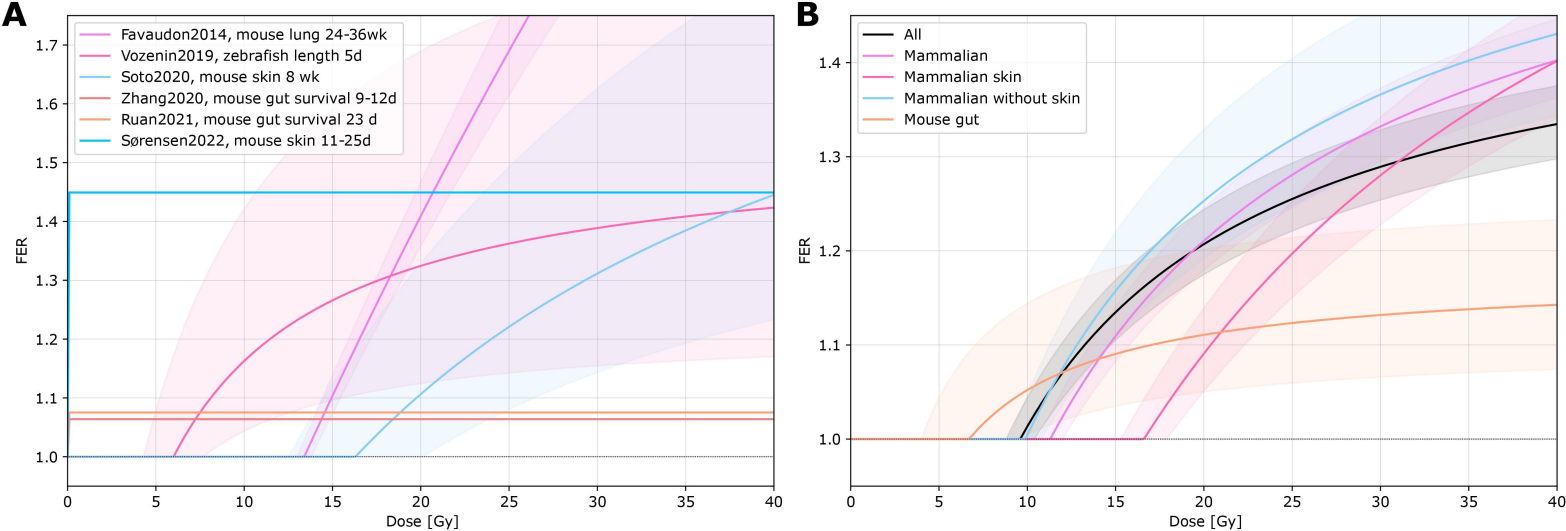
FER vs. dose. The fit function and function parameters (dose threshold and maximal sparing effect) for the individual (from a single dataset) and pooled data series were derived from Böhlen et al. (3). **(A)** Individual data series fits (Favaudon, 2014 (10), Vozenin, 2019 (14), Soto, 2020 (45), Zhang, 2020 (46), Ruan, 2021 (15), Sørensen, 2022 (19)). **(B)** Pooled data series fits of 27 datasets. The data were pooled into different sub-groups (all data, mammalian data, mammalian skin data, mammalian data without skin, and mouse gut data).

One systematic review by Böhlen et al. (3) was identified for the quantification of dose. They analysed 27 experimental datasets. The dose dependence of normal tissue sparing was parametrized for individual data (10, 14, 15, 19, 45, 46) from a single dataset or for pooled data grouped into all data, mammalian data, mammalian skin data, mammalian data without skin, and mouse gut data. Different tissues, endpoints, animal species, and beam types (e.g. electrons or protons) were included. The dose threshold and maximum sparing effect were determined in the review, their results were converted to dose-FER curves as seen in **Figure 3** for the individual (A) and pooled data (B). Results showed a variable dose threshold, if any was determined, and a variable maximum sparing effect. A smaller effect was found for mouse gut (maximum FER = 1.18 ± 0.09) compared to skin or lung data (maximum FER = 1.96 ± 0.12 for skin data, maximum FER = 1.49 ± 0.05 for all pooled data). With the creation of the different sub-groups, the sensitivity to the investigated tissue types was reduced. In the low-dose region, data are lacking.

**Figure 4:**
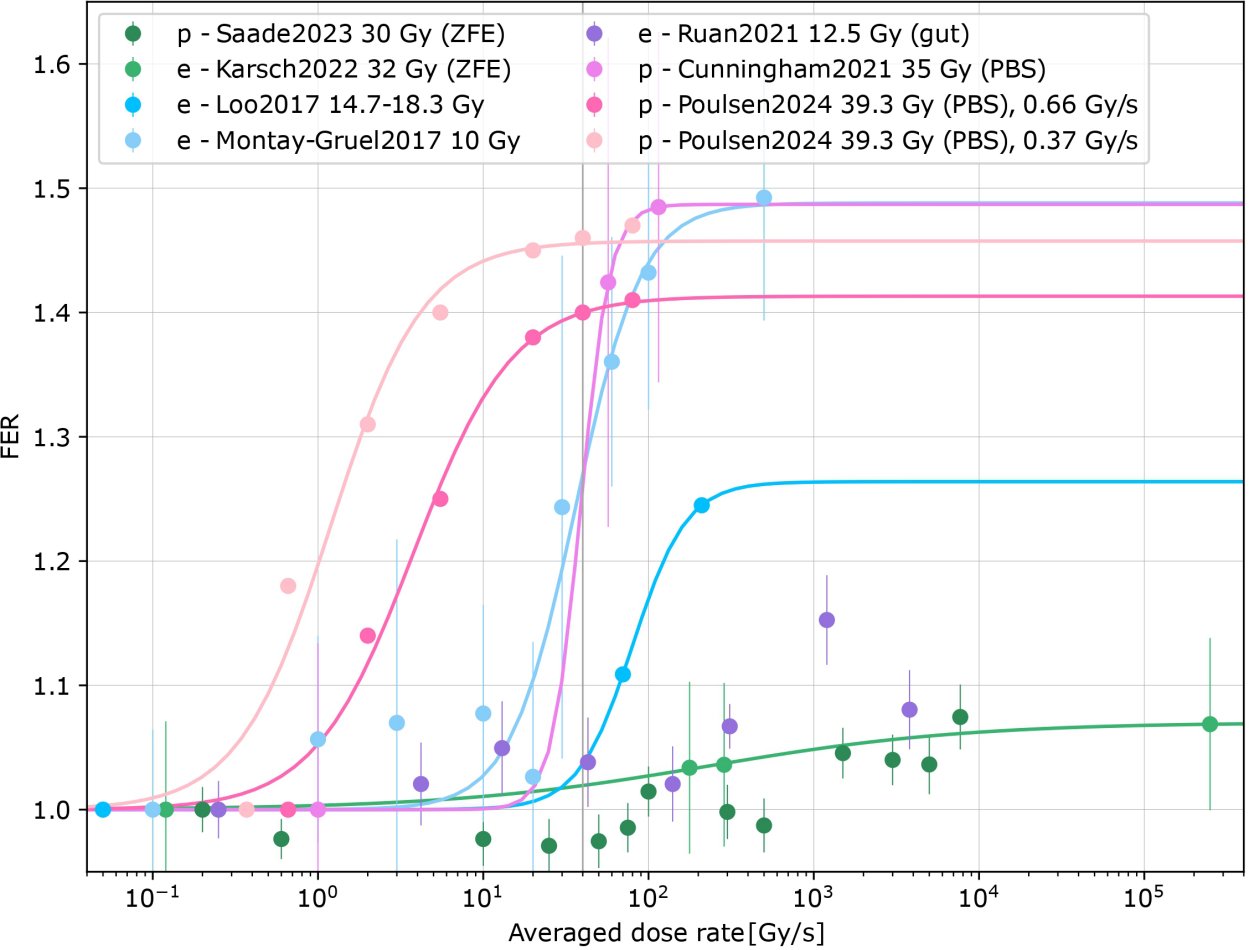
Converted FER vs. averaged dose rates for eight datasets [Saade, 2023 (47), Karsch, 2022 (8), Loo, 2017 (13), Montay-Gruel, 2017 (11), Ruan, 2021 (15), Cunningham, 2021 (20), Poulsen, 2024 (48)] for eFLASH (e) and pFLASH (p) for zebrafish embryos (ZFE) or mice. Proton irradiations with pencil beam scanning (PBS) are specified in the figure. Logistic fits for datasets with significant differences were added [exceptions: Ruan2021 (15) and Saade2023 (47)].

Seven records quantified the dependence of the FLASH effect on dose rate (8, 11, 13, 15, 20, 47, 48) (**Supplementary Table S3** in the **Supplementary Material**). Different tissues, endpoints, animal species, beam types and dose prescriptions were included. The dose rate dependence was parametrized by the maximum FER, midpoint, and steepness. The maximum FER ranged between 1.07 and 1.49, the midpoint dose rate value from 0.2 Gy/s to 5.5 Gy/s, the steepness parameter from 0.6 to 5.1 s/Gy. This variability prohibited further synthesis, with additional confounds due to different dose prescriptions and species as well as beam types. Across datasets, maximum sparing was reached within 25–250 Gy/s (**Figure 4**), also depending on the conventional DR used for comparison. The data suggest a saturation of the FLASH effect at high dose rates. At intermediate DR, reduced sparing was observed throughout the data, except for non-significant findings for mouse gut (15) and zebrafish embryos (47).

The effect of beam pauses on the FLASH effect was investigated in 4 records (15, 48–50), with one only providing a qualitative description (49). Two pFLASH datasets (48, 50) followed a similar protocol and used total doses of 30–40 Gy with 2-minute pauses on mice skin and muscle, respectively. A reduced sparing effect was observed with an increasing number of pauses at the same total dose, i.e. FER = 1.44-1.61 for a single delivery, 1.18-1–28 with one pause and 1.07-1.10 for two pauses. One eFLASH dataset (15) used two pulses and pauses ranging from milliseconds to seconds on mouse gut. The results were non-significant for the 30–31-week-old mice. For the 9–10-week-old mice, a decreasing sparing effect was observed with increasing pauses (CONV DR is not available for comparison). Since pFLASH studies differed in their timing setup from the eFLASH, the results were not synthesized further. The qualitative dataset (49) comparing the histology of lungs observed no differences between a single delivery and a delivery of 20 Gy split into 10 beams separated by 1 min. For further details on the extracted datasets, see **Supplementary Table S4** in the **Supplementary Material**.

**Figure 5:**
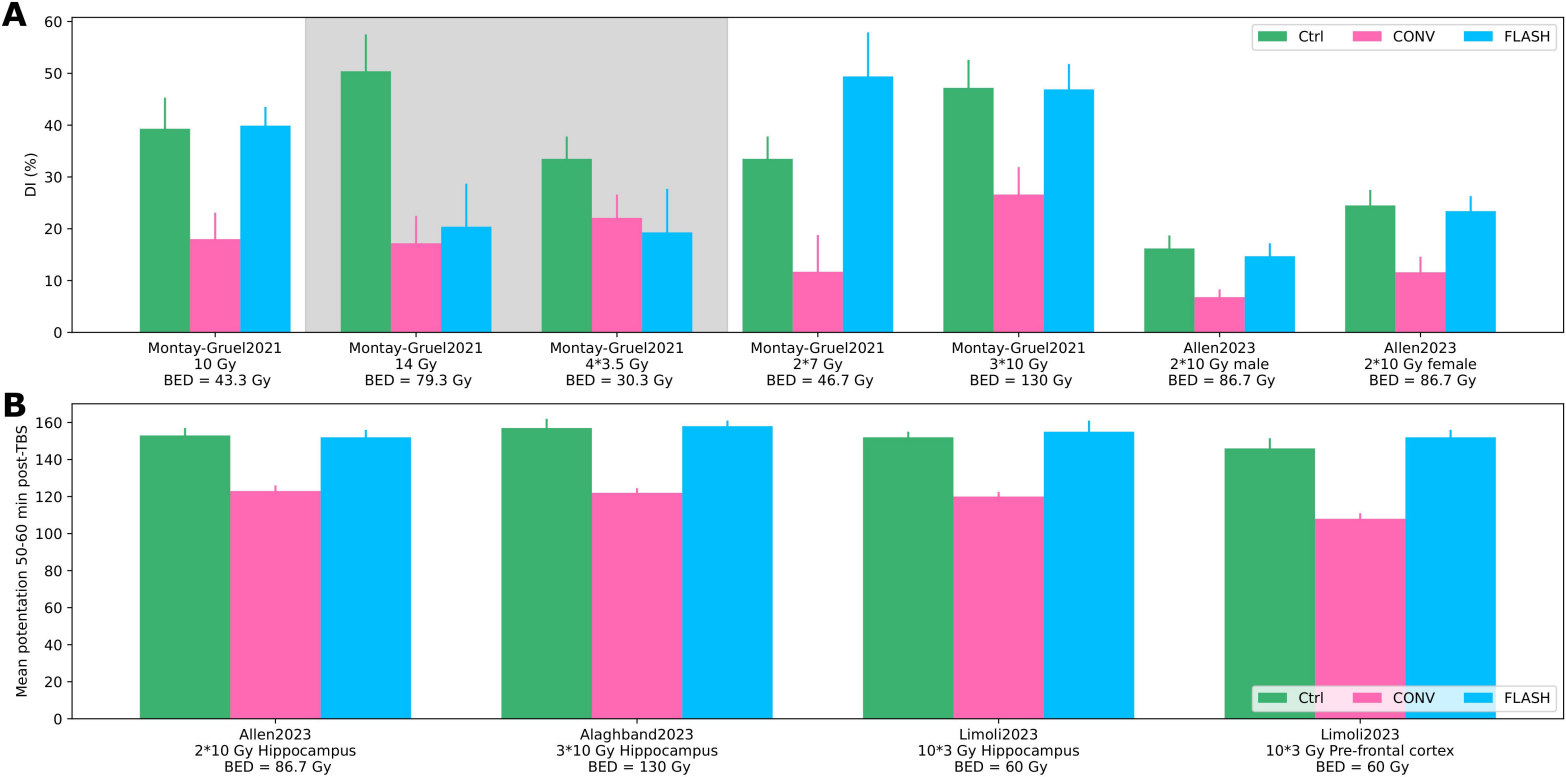
Fractionation. **(A)** Discrimination index from novel object recognition test for different fractionation schemes and biologically effective dose (BED) for brain (α/β = 3) for the control, CONV, and FLASH group from Montay-Gruel, 2021 (54) and Allen, 2023 (52). Shaded areas show no significant differences between CONV and FLASH. **(B)** Electrophysiology (mean potentiation 50–60 min after theta burst stimulation) for the control, CONV, and FLASH group from Allen, 2023 (52), Alaghband, 2023 (51), and Limoli 2023 (53).

Fractionated dose deliveries were extracted from four records (51–54), all using mice brain function to assess different (hypo-)fractionated schedules (**Supplementary Table S5** in the **Supplementary Material**). Novel object recognition and electrophysiology tests were used (see **Figure 5**). No difference was observed between the control and FLASH groups alongside a significant reduction of brain function for the CONV group for one to three fractions of 10, 2 × 7, and 10 × 3 Gy across the presented tests. However, the FLASH effect was lost at a single delivery of 14 Gy; no significant results were reached at 4 × 3.5 Gy. As the fractionation scheme differed for each experiment, the results were not synthesized further.

Because only positive results were included for the quantification of the physical FLASH conditions, the results might be biased toward a larger FLASH effect. In addition to the selection of reports, a publication bias that favors positive results regarding the FLASH effect might be present. Moreover, as the overall body of literature is small, the overall certainty in the body of evidence could also be regarded as small.

#### 3.1.2 Treatment planning studies

**Figure 6:**
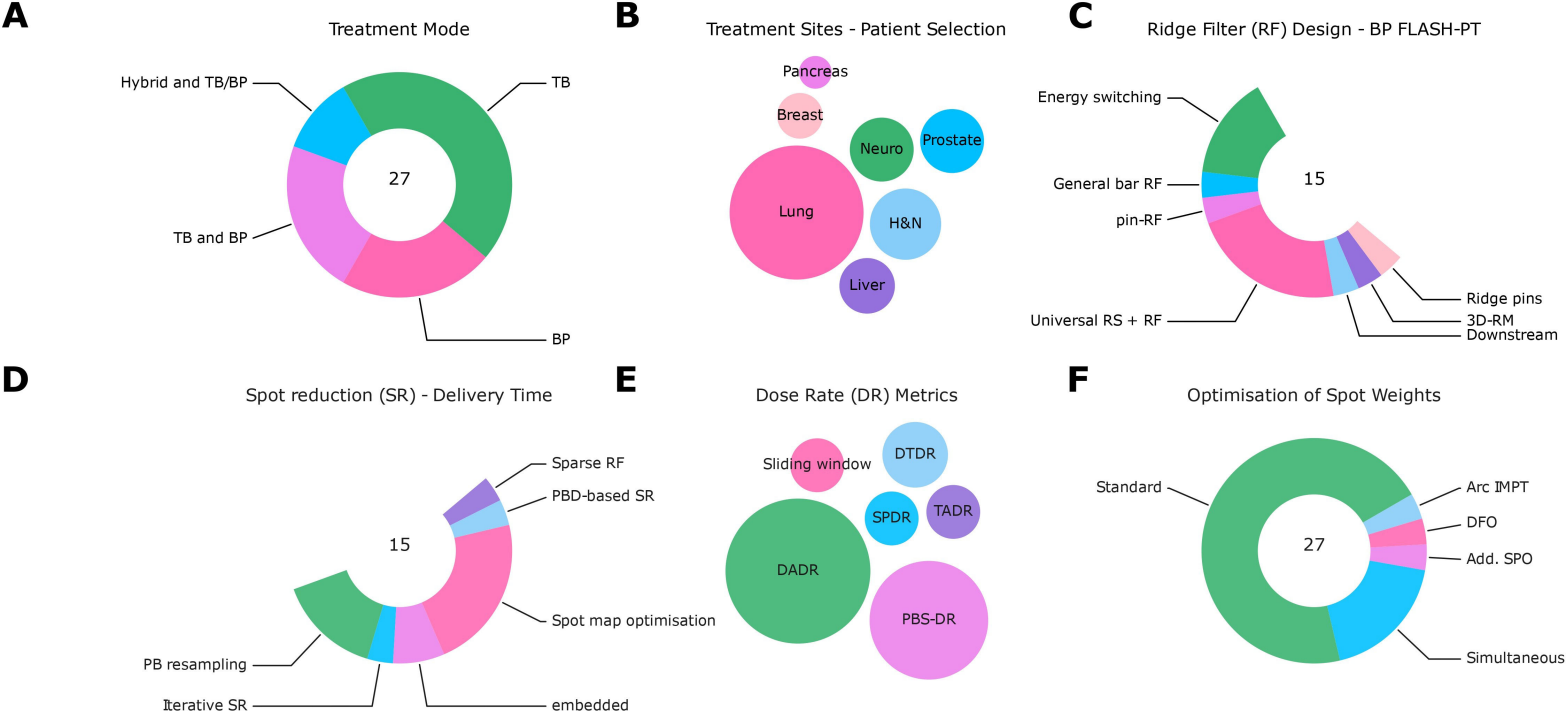
Summary of data analysis from extracted treatment planning studies. **(A)** Treatment modes (TB: transmission beams; BP: Bragg peak beams; TB and BP; Hybrid and TB/BP). **(B)** Treatment sites [pancreas, breast, lung, neuro, prostate, head and neck (H&N), and liver]. **(C)** Ridge filter designs [concepts: general bar RF (66), pin-RF (24), universal range shifters and range compensators (68), ridge pin designs (75), 3D-RM (23)]. **(D)** Spot reduction (SR) algorithms [concepts: spot map optimization (68, 72), PB resampling (76, 77), iterative spot reduction (66), pencil beam direction (PBD)-based spot reduction (24), the design of sparse RF or embedded (67, 74, 75)]. **(E)** Dose rate (DR) metrics [concepts: sliding window (25), dose threshold DR (57), spot-peak DR (26), time-averaged DR, PBS-DR (78), dose-averaged DR (DADR) (27)]. **(F)** Optimization algorithms of spot weights [concepts: standard, simultaneous (55, 65, 75), disjoint fields (DFO) (25), additional scan pattern optimization (SPO) (63), arc IMPT (74)].

Data from 27 records (24–29, 55–75) are summarized in **Supplementary Table S6** in the **Supplementary Material**, the main results of the analysis with respect to solutions and mitigation strategies for FLASH-PT are shown in **Figure 6**.

The ridge filter (RF) design for BP FLASH-PT to reduce overall delivery time followed different approaches: part of the studies relied on conventional energy switching or downstream modulation (29); general bar RF (66), pin-RF (24), universal range shifters and range compensators (68), ridge pin designs (75), and 3D-RM (23) were also used. Dose calculation was facilitated by ray-tracing or pre-calculating the ridge filters for different pin heights (see **Supplementary Table S6** for more information).

Different methods for spot reduction to reduce the overall delivery time have been tested: spot map optimization (68, 72), PB resampling (76, 77), iterative spot reduction (66), pencil beam direction (PBD)-based spot reduction (24), the design of sparse RF, or other reductions that are embedded into optimization algorithms (67, 74, 75).

Various DR metrics were used: sliding window (25), dose threshold DR (57), spot-peak DR (26), time-averaged DR, PBS-DR (78), and dose-averaged DR (DADR) (27). In several studies, various definitions were used for the evaluation; the heterogeneity reflected the uncertainty about the most suitable definition.

While most studies used conventional optimization approaches (single/multi-field optimization, single-field uniform dose or IMPT), others proposed novel approaches: simultaneous dose and DR optimizations (55, 65, 75), objectives to optimize for disjoint fields (DFO) (25), a sequential approach to optimize the scan patterns after dose optimization (SPO) (63), or other approaches (arc IMPT) (74). Dose rate optimization relied on different DR metrics such as DADR and PBS-DR.

Delivering a single field per fraction as a subset of a multi-field plan (alternating fields) was proposed to ensure the technical deliverability of FLASH proton therapy (56) or to circumvent and mitigate uncertainties about the effect (28). Transmission beams were tested with a varying number of fields, with too few fields resulting in unacceptable plan quality with respect to dose conformity (59, 61). With spot spacing, a trade-off between plan quality and FLASH-dose was seen (62, 64). Evaluation tools include dose rate volume histograms and dose rate distributions. Furthermore, irradiation times, number of irradiations, and metrics such as the FLASH coverage were used. Others used the FLASH-modified dose based on FER or the FLASH effectiveness model (29).

In the selected reports, a variety of approaches were present in each category, which might not be attributed to reporting biases and lack of certainty in the body of evidence but rather reflected the explorative and advancing developments in FLASH-PT treatment planning and delivery.

### 3.2 Impact assessment

**Figure 7:**
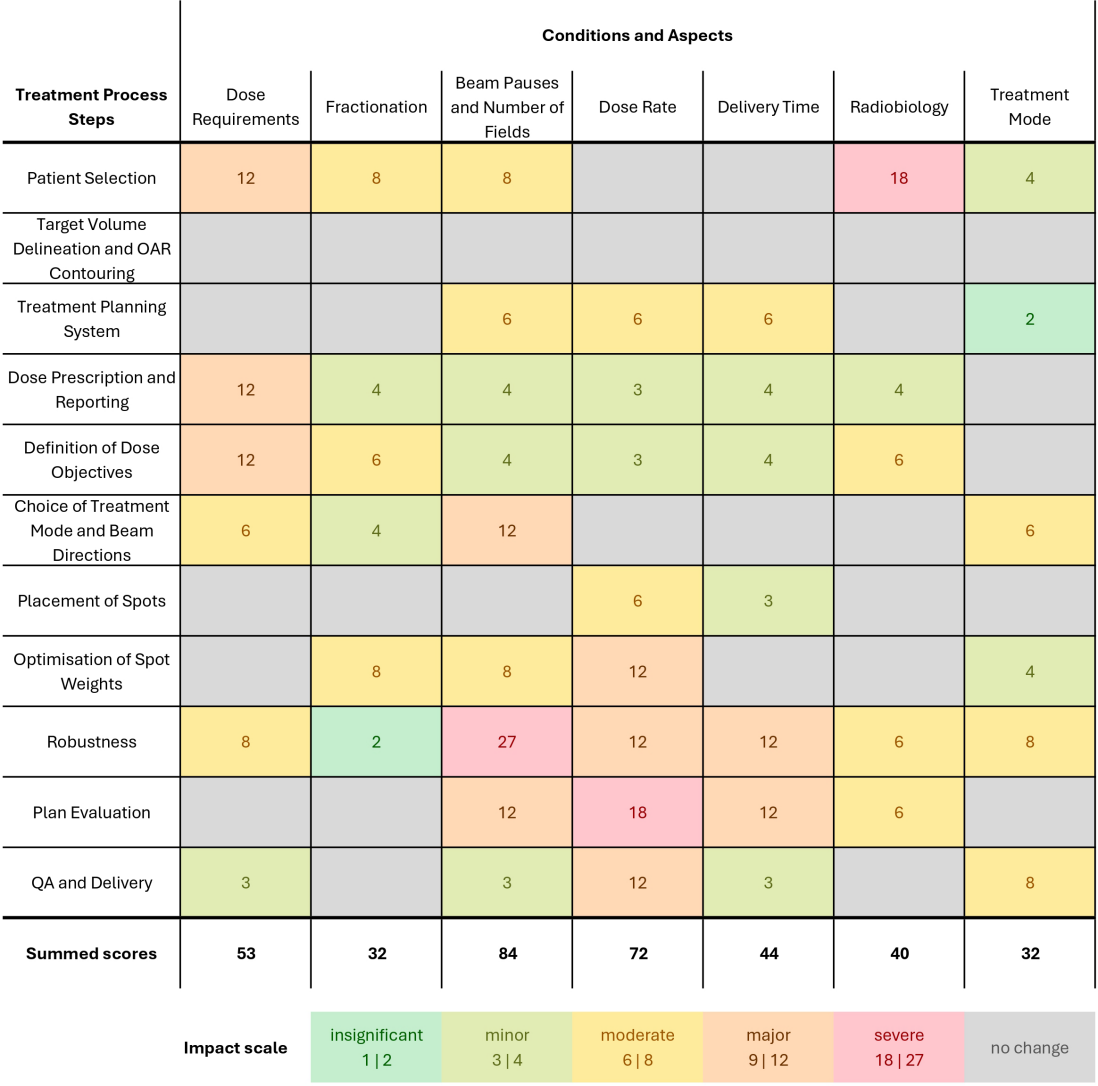
Impact assessment of the treatment process for FLASH-PT for near-future applications for physical FLASH conditions (dose, fractionation, beam pauses and number of fields, dose rate), delivery-related requirements (delivery time, treatment mode to account for transmission and BP beams), and the requirements to account for radiobiology. For each process step and FLASH condition, (i) the available knowledge, (ii) the criticality for clinical application, and (iii) the proposed mitigation strategies and solutions were scored on a three-point scale [1–3]. A higher score indicates more critical, less understood, and less mitigable aspects. Then, five impact levels were assigned by multiplying the sub-scores, ranging from insignificant impact not affecting clinical routine to severe impact causing severe complications for clinical translation. The summed scores for each condition were used to describe the overall impact.

The results of the impact assessment on clinical FLASH-PT are shown in **Figure 7**. The information from the review was used to inform the knowledge and mitigation strategies’ scores and combine it with what was relevant in each step of the treatment process. The overall order of importance for the FLASH conditions and aspects did not change before a consensus was reached. The sub-scores for criticality, knowledge, and mitigation strategies—that were reviewed in a discussion about each intersection—were equal for 101 of 144 sub-scores; the remainder deviated by one point from another in the individual scoring. All deviations were discussed to reach a consensus. The individual sub-scores are reported in **Supplementary Tables S9–S11** in the **Supplementary Material**.

Different impact levels were reached for various pairs of FLASH conditions and process steps. An insignificant and minor impact only calls for small adjustments in clinical routine, while a major and severe impact results in substantial modifications to either mitigate uncertainties or ensure the deliverability.

No impact was found for the target volume delineation and OAR contouring step. The total sum of impact scores for each parameter is highest for the beam pauses, followed by dose rate. Dose requirements, i.e., a potential minimum dose, often have a major impact due to their criticality for all clinical applications and limited knowledge and mitigation strategies. The scores for the fractionation condition are lower due to their reduced criticality and available mitigation strategies, i.e., avoid fractionation in near-future applications. Beam pauses and the number of fields are highly critical in the choice of beam directions and in robustness, and limited or no knowledge and mitigation strategies may result in a major to severe impact of these. A similar pattern was observed for the dose rate requirement at steps with a major or severe impact. A radiobiological understanding is highly critical throughout most treatment planning steps due to the possible clinical consequences of changes in the biological effect but often has a minor to moderate impact due to the possibility to circumvent the uncertainties. Treatment mode alterations with respect to TB or BP beams produced from ridge filters offer knowledge and solutions for FLASH-PT, resulting in moderate to insignificant impact.

## 4 Discussion

We conducted an impact assessment of several FLASH conditions on the FLASH-PT treatment process. To this end, we identified (i) the currently available knowledge and gaps therein, (ii) the criticality for several aspects of clinical application, and (iii) mitigation strategies and solutions based on data extracted from a systematic literature review. In accordance with the FLASH conditions defined above, our main findings are discussed below.

Substantial uncertainty remains with respect to the lower and upper limits of dose (in one fraction) for the FLASH effect (3), which has a major impact on the selection of treatment sites and dose to tumor and OAR. The lack of data for lower doses and the pre-clinical setup using single doses on small volumes further introduces uncertainty of its clinical translatability.

Since fractionation may have a differential effect on the FLASH effect beyond fraction dose thresholds, it is considered as an independent condition. However, limited pre-clinical data (51–54) constrains fractionation schemes compatible with both FLASH and clinical applications. To circumvent uncertainties about fractionation, single-fraction or hypo-fractionated treatments have been proposed (4, 79).

Multiple beams allow for robust and conformal treatment plans but introduce pauses during the delivery of one fraction. Beam pauses on the order of tens of seconds to minutes emerge as the most crucial parameter for treatment planning and delivery due to their relevance for multiple fields and for treatment interruptions. Limited knowledge about re-irradiation during one fraction (15, 48–50) restricts the number of fields; the application to more complex sites or the use of transmission beams becomes infeasible with respect to dose conformity. Limited knowledge about beam interruptions adds a severe safety concern to the delivery of FLASH-PT plans.

Various studies (8, 11, 13, 15, 20, 47, 48) support a minimum average dose rate for an optimal FLASH effect with electrons and protons. Various dose rate definitions have been proposed to account for temporal and spatial aspects of dose delivery with PBS. However, it is unknown which serves as the best predictor of the FLASH effect (80). This has a major impact on the optimization and evaluation steps. Machine information such as beam current for the calculation of dose rate in the TPS and a robust approach regarding delivery-related fluctuations have to be integrated into the planning process.

To decrease delivery time, spot placement and reduction (24, 66–68, 72, 74–77) are critical for the deliverability of PBS FLASH-PT, as the reduction of spots is likely to increase the dose rate (PBS-DR).

Radiobiological unknowns about the magnitude of the normal tissue sparing by the FLASH effect across tissues or patients can be safely circumvented in most planning steps by staying within conventional constraints on physical dose. In this way, it may be considered as an added benefit in the evaluation of a clinically acceptable plan. However, the lack of mechanistic understanding on both normal tissue and tumor response has a severe impact on the patient selection step and creates barriers for clinical translation.

Transmission beams come at the disadvantage of dose distal to the target and decreased dose conformity, which limits beam directions and treatment sites. In contrast, using the plateau region reduces range uncertainties, making it feasible for, e.g., lung treatments (81). Additionally, transmission beams (64) or dedicated algorithms (75) circumvent LET uncertainties. For BP FLASH-PT, the ridge filter design, optimization, and robustness (manufacturing, positioning, plan adaptation), evaluation, and QA need to be constructed for this new planning and delivery process.

For QA and delivery of dose, dose rate, and beam pauses, sub-millisecond resolution is required. Protocols and detectors are being developed and tested for UHDR conditions; the temporal characteristics could also be extracted from machine log files (33, 34). Some process steps such as delineation and contouring require no or little changes for FLASH-PT or offer knowledge and mitigation strategies that reduce the impact of biological uncertainties and treatment mode aspects.

The characterization of physical conditions using the extracted pre-clinical data is limited by the current body of literature, uncertainties in the datasets, the FER conversion, and assumptions about the FLASH effect, e.g., the independence of particle type (5–9) and a saturation above 40–100 Gy/s and no sparing for the baseline. Furthermore, the inhomogeneity of the datasets with respect to the delivery of single (passively scattered) beams or PBS (20, 48, 50) is not reflected in the average dose rates used for analysis. A synthesis of individual results aside from the dose condition (3) is not feasible yet due to lack of data (3). Aspects relevant for the clinical translation, such as using lower doses (per fraction), multiple fields, considering larger volumes, and different targeted tissues, cannot be fully derived from pre-clinical studies.

The extracted treatment planning studies followed various UHDR-compatible approaches. As FLASH-PT planning and delivery is still in the early development stage, it concentrated on presenting new algorithms and proof-of-principle mitigation approaches and solutions rather than demonstrating clinical feasibility for a selected patient group. Some approaches (26, 56–59, 61, 62, 68, 69, 71, 72) have been used in multiple treatment planning studies by the same research group. Another limitation in the reviewed studies can be regarded as the continuous development and incorporation of gained knowledge on FLASH-PT planning and delivery, which limits the use of initial approaches.

During the review process, we mainly relied on single screening, risk of bias rating, and data extraction, which introduced the risk of error. Nevertheless, the plausibility of decisions was discussed with a second author; the subsequent impact assessment was performed dually and independently. Furthermore, only a small number of records with large variability were included in the final analysis, which may further reduce the validity of the results. Ultimately, the confidence in the overall conclusions is not expected to change due to these methodological limitations. As more data becomes available, the methodology can be revisited. Similarly, tumor control studies could be added in future analyses.

The impact assessment effectively integrates available information of the FLASH effect with the treatment process steps and was used to evaluate the status of the clinical development of the modality. Key focus areas—available knowledge and gaps, criticality for clinical applications, and solutions and mitigation strategies for its incorporation—were scored for different FLASH conditions and process steps. However, dependencies may exist between different process steps due to the proposed solutions and mitigation strategies for FLASH-PT. Furthermore, there might be unknown dependencies and connections that cannot be fully accounted for. Mitigation strategies for beam pauses such as alternating fields (4, 28) or disjoint fields (25) come at the expense of normal tissue sparing through fractionation or robustness against uncertainties, respectively. Apart from optimization algorithms, dose rates can also be increased by determining an optimal spot spacing or disabling repainting (48) at the expense of dose homogeneity and robustness against (breathing) motion, respectively. As another example, hypofractionation potentially leads to a different impact of setup uncertainties (82), and single fields do not allow to mitigate RBE uncertainties. Depending on the choice of treatment mode and treatment indication, the individual impact may differ.

The considerations in plan generation with respect to dose, fractionation, beam pauses, and the mechanistic aspects may be generalizable to other treatment modalities; for example, very-high-energy electrons (VHEE) (83) as the link between those FLASH conditions and planning steps are not specific to FLASH-PT delivery characteristics. Meanwhile, treatment planning steps such as optimization, evaluation, and QA relate to the delivery characteristics of PBS PT as well as practical machine limitations, e.g., on achievable and safe dose rates.

The FLASH effect was demonstrated for both plateau and BP (84, 85) irradiations pre-clinically, with higher dose rates available in the BP and less tolerable dose observed at same dose rates in the BP (9). Few applications for transmission beams are foreseeable and were tested in treatment planning studies, but the technical limitation to achieve UHDR with BP beams was overcome with hardware developments. The decreased delivery time may offer the ability to “freeze” tumor and organ motion, which has great potential to reduce uncertainties in conjunction with motion management strategies (34). FLASH-PT may further offer opportunities to expand and safely use hypofractionation (2).

Radiobiological modeling is based on mechanistic hypotheses (35), phenomenological observations (29, 86), or a combination of both (48). Models can be integrated first as an evaluation tool and be refined based on pre-clinical and clinical data to enable their use in plan generation and optimization. Parameters like dose, timing, and machine-related parameters should be adaptable and integrable. The modeling, optimization, and evaluation of the FLASH effect with respect to dose rate requires a definition relating the delivery timing to corresponding radiobiological evidence [e.g., field dose rates of around 80 Gy/s correspond to a PBS-DR_95%_ of 185 Gy/s with total delivery times of up to 730 ms in one pFLASH study using PBS (19)].

Uncertainties about the FLASH effect with respect to dose and delivery dynamics—dose rate and timing—require further pre-clinical studies for a more complete characterization. Low doses and fractionated treatments may be investigated in conjunction for different tissue types. Most relevant for clinical translation, however, is the effect of beam pauses or split doses within a fraction and how the FLASH effect dynamics evolve from its observed effect at high dose rates to multiple re-irradiations within one fraction to the tissue recovery observed for fractionated deliveries. Systematically varying the duration of the beam pauses and the magnitude and number of split doses could provide further insights needed when using small fraction doses or multiple fields in one fraction.

Gaining evidence about normal tissue sparing and tumor control in humans will also help to decrease radiobiological knowledge gaps. Palliative or oligo-metastatic settings could facilitate safe application for early FLASH-PT. Large fraction doses and single fields with either transmission or BP beams could already be tested in these clinical indications. Future treatment planning studies should maintain their focus on the development and integration of solutions for FLASH-PT while also considering the potential of clinical treatment planning and delivery for selected treatment sites with respect to deliverability, safety, plan quality, and robustness. Furthermore, exploring the possibilities to combine FLASH and conventional IMPT or boosting the FLASH effect in certain regions might be considered, especially with respect to larger target volumes.

In summary, the integration of the FLASH effect into clinical proton therapy is already foreseeable in some clinical applications but requires the extension of planning, delivery, and uncertainty mitigation strategies. This includes the development of modular and adaptable treatment planning systems and parameter sensitivity analyses to ensure robust planning despite the remaining FLASH uncertainties. Biological unknowns about the dose threshold and delivery timing (fractionation, beam pauses, and dose rate) hinder the adequate understanding and modeling of the FLASH effect, which is crucial for FLASH-PT planning and delivery strategies for a wider range of clinical applications.

## Data Availability

The original contributions presented in the study are included in the article/**Supplementary Material**. Further inquiries can be directed to the corresponding author.
