# Supplementary material for: Impact assessment of current knowledge gaps and mitigation strategies in clinical FLASH proton therapy through a systematic review
Source: Front Oncol. 2025 Sep 11;15:1550264. doi: 10.3389/fonc.2025.1550264 (PMC12460130; doi:10.3389/fonc.2025.1550264)
Supplement: Supplementary file 1 [file DataSheet1.pdf]

Impact assessment of current knowledge gaps and mitigation strategies in clinical FLASH proton therapy through a systematic review

Supplementary Material

Literature search

Table 1: Overview of searched databases performed by the Medical Library of Erasmus MC. Database and database platform, their years of coverage, found records in each database and records after duplicates were removed.

**Database searched**	**Platform**	**Years of coverage**	**Records**	**Records after duplicates removed**
Medline ALL	Ovid	1946 - Present	702	689
Embase	Embase.com	1971 - Present	1167	532
Web of Science Core Collection*	Web of Knowledge	1975 - Present	1369	577
Cochrane Central Register of Controlled Trials**	Wiley	1992 - Present	38	20
Additional Search Engines: Google Scholar***			200	49
**Total**			3476	1867

* Science Citation Index Expanded (1975-present); Social Sciences Citation Index (1975-present); Arts & Humanities Citation Index (1975-present); Conference Proceedings Citation Index- Science (1990-present); Conference Proceedings Citation Index- Social Science & Humanities (1990-present); Emerging Sources Citation Index (2005-present)
*Exact search turned on in Web of Science Core Collection
** Manually deleted conference abstracts from fore 2021.
***Google Scholar was searched via "Publish or Perish" to download the results in EndNote.

No other database limits were used than those specified in the search strategies

Table 2: Search strategy in the databases for FLASH articles.

**Database**	Search terms
Medline	((((flash OR UHDR OR ultra-high-dose-rate OR ultrahigh-dose-rate) ADJ4 (radiation* OR radiotherap* OR radio-therap* OR RT OR proton* OR irradiat* OR beam* OR ion OR particle* OR therap*)) OR flash-effect*).ab,ti,kf.) AND 2014:2030.(sa_year) NOT ((news OR congres* OR abstract* OR book* OR chapter* OR dissertation abstract*).pt. AND 1800:2021.(sa_year))
Embase	('FLASH radiotherapy'/exp OR (((flash OR UHDR OR ultra-high-dose-rate OR ultrahigh-dose-rate) NEAR/4 (radiation* OR radiotherap* OR radio-therap* OR RT OR proton* OR irradiat* OR beam* OR ion OR particle* OR therap*)) OR flash-effect*):ab,ti,kw) AND ([2013-2030]/py) NOT ([Conference Abstract]/lim AND [1800-2021]/py)
Web of Science	TS=(((flash OR UHDR OR ultra-high-dose-rate OR ultrahigh-dose-rate) NEAR/3 (radiation* OR radiotherap* OR radio-therap* OR RT OR proton* OR irradiat* OR beam* OR ion OR particle* OR therap*)) OR flash-effect*) AND PY=2015-2030 NOT (DT=(Meeting Abstract OR Meeting Summary) AND PY=1800-2021)
Cochrane CENTRAL	((((flash OR UHDR OR ultra NEXT/1 high NEXT/1 dose NEXT/1 rate OR ultrahigh NEXT/1 dose NEXT/1 rate) NEAR/4 (radiation* OR radiotherap* OR radio NEXT/1 therap* OR RT OR proton* OR irradiat* OR beam* OR ion OR particle* OR therap*)) OR flash NEXT/1 effect*):ab,ti,kw)
Google Scholar	flash|UHDR|'ultra high dose rate'|'ultrahigh dose rate' radiation|radiotherapy|'radio therapy'|irradiation|RT|proton|beam|beams|ion<br><br>(year filter 2013-2030)

Data extraction – in-vivo pre-clinical data

Dose rate

After conversion, the individual datasets were fitted with a logistic function (see Equation S-1) if they produced significant results. The normal tissue sparing as a function of dose rate was estimated for each dataset.

$$F(\dot{D})_{\dot{D}_{50},\gamma_{50}^{\mathrm{FER}},\mathrm{FER}^{\max}} = \frac{\mathrm{FER}^{\max}}{1 + e^{-\gamma_{50}^{\mathrm{FER}}(\log(\dot{D}) - \dot{D}_{50})}} + 1 \qquad \text{(S-1)}$$

In an attempt to homogenise the datasets, the data was normalised to the maximum effect under the assumption that it is reached in each dataset. The pooled normalised data was fitted with a logistic function.

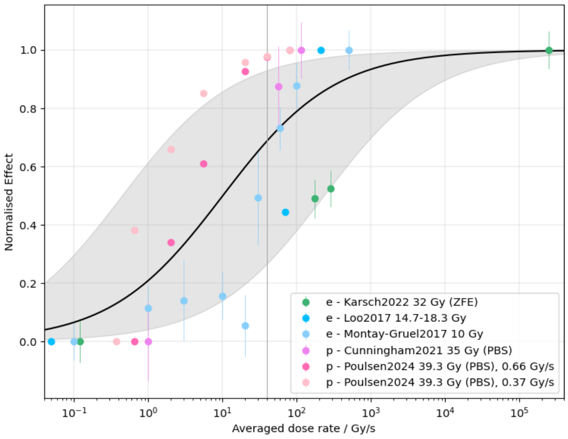

Figure A.1: Normalised effect vs. averaged dose rates for datasets with significant differences and logistic fit of pooled data.

Table 3: Pre-clinical studies comparing normal tissue sparing of UHDR and CONV irradiations with respect to the dose rate

Study	Biologic system, irradiated volume	Endpoint & assessment time	Beam type	Dose [Gy]	Time-averaged dose rate (TADR) [Gy/s]	Other UHDR characteristics, delivery time (Δt)
**(20) Cunningham2021**[#]<br>doi:10.3390/cancers13051012	C57Bl/6j mice (Leg)	Leg contracture (12 wk)<br>Skin toxicity (96 h)	p+ plateau (244/250 MeV)	35	1, 57, 115	UHDR Δt: 240-610 msec
**(8) Karsch2022**[*]<br>doi:10.1016/j.radonc.2022.05.025	Zebrafish (WBI)	Fish length (4 d)	e- (30 MeV)	32	0.12,<br>286.7 (*isochronous synchrotron (ICS)*),<br>177.2 (*synchro-cyclotron (SC)*),<br>2.5e5 (*ELBE max*)	Δt: 0.1-240 sec<br>*ICS*: Δt: 0.1 sec,1.3e6 bunches, bunch DR 4.4e6 Gy/s<br>*SC*: Δt: 0.164 sec, 4125 bunches, bunch DR 1.4e9 Gy/s<br>*ELBE max*: Δt: 0.3 msec, 3935 bunches, bunch DR 1.5e9 Gy/s
**(13) Loo2017**[**]<br>doi:10.1016/j.ijrobp.2017.02.101	C57BL/6J mice (Total abdomen)	Mouse gut – survival (20 d)	e- (20 MeV)	10-22	0.05, 70, 210	Δt: 0.05-0.31 sec
**(11) Montay-Gruel2017**[†]<br>doi:10.1016/j.radonc.2017.05.003	C57BL/6J mice (Whole brain)	Neuroprotection (novel object recognition) (2 mo)	e- (6/4.5 MeV)	10	0.1, 1, 3, 10, 30, 100, 500	UHDR pulse width: 1.8 μs<br>UHDR freq.: 100 Hz<br>Δt: 10 msec – 100 sec
**(48) Poulsen2024**<br>doi:10.1016/j.ijrobp.2024.02.050	CDF1 mice (Leg)	Skin toxicity (25 d)	p+ plateau (244/250 MeV)	39.3	0.7, 2, 5.5, 20, 40, 60, 80	
**(15) Ruan2021**[‡ §]<br>doi:10.1016/j.ijrobp.2021.08.004	C3H mice (Abdomen)	Mouse gut crypt survival (3.75 d)	e- (6 MeV)	12.5	0.25, 4.2, 13, 43, 140, 310, 1.2e3, 3.8e3, 3.7e6	UHDR pulse width: 3.4 us, UHDR per pulse: 0.01 - 3.7e6 Gy, UHDR freq.: 1-1250 Hz<br>Δt: 0.0034 ms – 50 sec

**(47) Saade2023** * § doi:10.1016/j.adro.2022.101124	Zebrafish (WBI)	Length (4 d)	p+ plateau (68 MeV)	30	0.2, 0.6, 10, 25, 50, 75, 100, 300, 500, 1500, 3000, 5000, 7700	Single pulse

[#]Pencil beam scanning (ProBeam)
[*] FER were derived from ratio of zebrafish lengths of UHDR irradiation vs zebrafish length at CONV irradiation
[**] Data from study that was not peer-reviewed
[†]FER was derived from ratio of recognition index of UHDR irradiation vs recognition index at CONV irradiation at 10 Gy
[‡]Maximal FER of 1.1 reported in the study used for highest UHDR irradiation with endpoint of 10% crypt survival (12.7 (12.1-13.4) Gy CONV and 13.9 Gy (13.3-14.7) Gy UHDR for 9 wk old mice; 30 wk old mice), intermediate dose rates scaled accordingly
[§]Not significant

Beam pauses

Table 4: Pre-clinical studies comparing normal tissue sparing of UHDR and CONV irradiations with respect to beam pauses. Total dose split into equal portions for beam pause investigation.

Study	Biologic system, irradiated volume	Endpoint & assessment time	Beam type	Dose [Gy]	TADR [Gy/s]	Other UHDR characteristics, delivery time Δt	Outcome
**(49) Dai2023** * doi:10.1080/09553002.2023.2194403	Balb/c nude mice (Whole thorax)	Lungs (72 h)	γ (1-2 MeV) UHDR 69Co CONV	20	0.033, 200	UHDR: 1 and 10 pulses<br>Time between pulses: 1 min<br>Dose: 1 and 10	No difference between split dose and single dose
**(50) Mascia2024** † doi:10.1016/j.ijrobp.2023.07.024	57Bl/6j mice (Leg)	Skin toxicity (7 wk)<br>Leg contracture (16 wk)	p+ plateau (244/250 MeV)	30, 35	1, 100	Δt: 0.3 sec to 4 min 0.35 sec<br>Time between pulses: 2 min<br>Dose splits: 1, 2 and 3	Reduction of FER with more splits<br>FER = [1.61, 1.18, 1.09] # 30 Gy<br>FER = [1.47, 1.28, 1.07] # 35 Gy
**(48) Poulsen2024** † doi:10.1016/j.ijrobp.2024.02.050	CDF1 mice (Leg)	Skin toxicity (25 d)	p+ plateau (244/250 MeV)	39.3	0.37, 60	Time between pulses: 2 min<br>Dose splits: 1, 2, 3, 4 and 6	Reduction of FER with more splits (at toxicity level of 2.5)<br>FER = [1.44, 1.23, 1.10, 1.10, 1.16]
**(15) Ruan2021** doi:10.1016/j.ijrobp.2021.08.004	C3H mice (Whole abdomen)	Mouse gut crypt survival (3.75 d)	e- (6 MeV)	11.2, 12,5	0.25/2.2 – 5.9e6	2 pulses with pulse interval from 3.3 msec to 30 seconds	Non-significant results for 30-31 wk mice.<br>9-10 wk mice with reduction of crypt survival at prolonged breaks (but no CONV as baseline)

* Only qualitative description: histopathological staining and tumour control.
† Pencil beam scanning

Fractionation

Table 5: Pre-clinical studies comparing normal tissue sparing of UHDR and CONV irradiations with respect to fractionation. All studies were performed for the same type of mice and looked at the same endpoints: the discrimination index of a novel object recognition test was used to test the cognitive skills of mice, and the mean potentiation as an electrophysiological measure (fEPSP slope - mean potentiation 50-60 min post-TBS). 48 hours between fractions

Study	Biologic system, irradiated volume	Endpoint & assessment time	Beam type	Dose [Gy]	TADR [Gy/s]	Other UHDR characteristics, delivery time Δt
**(51) Alaghband2023** doi:10.1158/2767-9764.Crc-23-0117	C57Bl/6J mice (Whole brain)	Long term potentiation, 6 mo; behavioural testing 4 mo	e- (6 MeV)	3x 10 Gy	0.9, 5.6e6	Single pulse UHDR pulse width: 1.8 μs
**(52) Allen2023** doi:10.1093/neuonc/noac248	C57Bl/6J mice (Whole brain)	Long term potentiation, 6 mo; behavioural testing 4 mo	e- (6 MeV)	2x 10 Gy	0.9, 5.6e6	Single pulse UHDR pulse width: 1.8 μs
**(53) Limoli2023** doi:10.1016/j.radonc.2023.109767	C57Bl/6J mice (Whole brain)	Long term potentiation, 6 mo	e- (6 MeV)	3x 10 Gy	0.9, 1.6e6	Single pulse UHDR pulse width: 1.8 μs
**(54) Montay-Gruel2021** doi:10.1158/1078-0432.Ccr-20-0894	Nude mice (Whole brain)	Behavioural testing (novel object recognition) (1 mo)	e- (6 MeV)	10, 14, 4x3.5, 2x7, 3x10, 25	0.1, >1.8e6	Pulse repetition frequency: 100 Hz Pulse width: 1.8 μs Δt: 1.9e-6 to 1e-2 sec

Treatment planning studies

Table 6: Treatment planning studies identified in systematic literature search. Treatment mode, ridge filter design, treatment (site, number of patients, prescription), number of fields, spot reduction, optimisation, dose rate (DR) definition, evaluation and other information relevant for FLASH-PT treatment planning

**Study**	**Treatment Mode**	**Ridge filter design**	**Treatment**	**Fields**	**Spot Reduction**	**Optimisation**	**DR Definition**	**Evaluation**	**Others:**
**(55) Gao2020** doi:10.1002/mp.14531	TB	-	Lung, 3 patients, 2/6/10 Gy/fx	1/3 beams for 2Gy, 3/5/9 beams for 6 Gy, 5/9/17 beams for 10 Gy, equiangular	-	Simultaneous Dose and DR Optimisation (SDDRO)	DADR	DRVH, dose rate distributions, DR coverage above 40 Gy/s	Motion management: Robustness: 5 mm setup, 5% range Comments: Hypofractionation for better DR coverage and potentially better FLASH effect, more beams improve dose distribution
**(26) vanMarlen2020** doi:10.1016/j.ijrobp.2019.11.011	TB	-	Lung, 7 patients, 54 Gy in 3 fx (SBRT)	10 largely non-coplanar fields for conformity	-	MFO	Spot peak DR	Dose rate, irradiation time, number of irradiations	Motion management: ITV from averaged 4DCT Robustness: Comments: 0.1 Gy threshold for #irradiations
**(56) vanMarlen2021** doi:10.3390/cancers13081859	TB	-	H&N, 10 patients, 35 fx of 2/1.55 Gy	10 coplanar, equiangular	-	MFO	Spot peak DR	Dose rate, beam-on irradiation time	Robustness: Comments: Alternating fields to ensure deliverability
**(57) Kang2021** doi:10.3390/cancers13143549	TB	-	Lung, 9 patients, 45 Gy in 3fx, 34 Gy in 1 fx	5 fields, equiangular	-	IMPT	ADR (PBS-DR, DADR, DTDR	DRVH	Motion management: Robustness: Comments: reduce minimum spot time or number of fields,

									inferior plan quality
**(58) Wei2021a** doi:10.3389/fonc.2021.813063	TB	-	Liver, 7 patients, 67.5 Gy in 15 fx (SBRT)	2/5 fields	-	SFO or MFO	ADR (PBS-DR)	DRVH, dose rate distributions	Motion management: ITV from averaged 4DCT Robustness:
**(59) Wei2022c** doi:10.3389/fonc.2022.970602	TB	-	Lung, 10 patients, 34 Gy in 1 fx (SBRT)	3/5 fields	-	MFO	ADR (PBS-DR)	V_40Gy/s for perturbation scenarios (max min bandwidth), DRVH etc.	Motion management: ITV from averaged 4DCT Robustness: 5 mm setup, 3.5% range, Comments: minimum MU per spot limits flexibility, 3 fields inferior, narrow V_40Gy/s bandwidths for perturbation scenarios, good FLASH coverage
**(60) Gao2022** doi:10.1002/mp.15356	TB	-	Lung (12 Gy in 3 fx), Brain (16 Gy in 1 fx), Prostate (12 Gy in 3 fx), H&N (15 Gy in 4 fx)	3 beams lung/brain, 2 beams prostate, 4 beams H%N	-	SDDRO	DADR	DRVH, dose rate distributions , V_40Gy/s	Motion management: Robustness: Comments: FLASH coverage above 8 Gy and 40 Gy/s
**(28) Habraken2022** doi:10.1016/j.radonc.2022.08.015	TB	-	Lung, 12 patients,	All or single beams in 1 fx, co-planar equiangular	-	SFUD		EQD2 with FER from 1 to 2 in 0.1 steps	Motion management: Robustness: Comments:

			54 Gy in 3/5/7/9 fx (SBRT)						SBPF loss of fractionation substantial but might be feasible
**(61) vanMarlen2022** doi:10.1016/j.adro.2022.100954	TB	-	Lung, 7 patients, 34 Gy in 1 fx (SBRT)	3/5/10 beams	-	MFO	DADR, PBS-DR, ADR (TADR)	FLASH-modified dose	Motion management: ITV from averaged 4DCT Robustness: 5 mm setup, 3% range Comments: 3 beams insufficient, sensitivity analysis for different dose and DR thresholds, beam current
**(62) vanMarlen2023** doi:10.3390/cancers15092579	TB	-	Breast, 3 patients, 2x9.74 Gy, 1x14.32 Gy	Single field	-	SFO	PBS-DR and sliding window, ADR (TADR)	FLASH-modified dose	Motion management: Robustness: Comments: 4 to 10 mm spot spacing, spot spacing trade-off quality and FLASH-dose; split fields (FLASH/non-FLASH)
**(63) JoseSanto2023** doi:10.1016/j.ijrobp.2022.08.053	TB	-	Lung, 20 patients, 54 Gy in 3 fx	3 beams, equiangular	Pencil beam resampling	SFUD and Scan Pattern Optimisation	PBS-DR	Dose rate distributions , FLASH coverage	Motion management: Robustness: Comments: Scan pattern optimisation, FLASH evaluated for each beam separately,

									above 8 Gy and 40 Gy/s, variable beam current
**(64) Kneepkens2023** doi:10.1088/1361-6560/ad0280	TB	-	Neuro, 5 patients, 28-33 fx of 1.8 Gy	5 fields, non-parallel opposed	-			LETd with FER of 1.5	Robustness: set up and range vox min max, shoot through avoids LET and range uncertainties Comments: Minimum spot distance 4 mm for UHDR feasibility
**(65) Lin2021** doi:10.1088/1361-6560/ac02d8	Hybrid	-	Lung, 3 patients, 6 Gy per fx	3 beams	-	Sampled as in IMPT for BP and SDDRO for TB = SDDRO-joint	DADR	Dose rate distributions , DRVH, DR coverage above 40 Gy/s	Motion management: Robustness: 5 mm and 5% robustness
**(66) Ma2023a** doi:10.1002/mp.16370	Hybrid and TB	*General bar RF* (Bar ridges on a base; bars formed of several steps and equally spaced on base; Pre-calculation of 9 bar RF with water equivalent heights (3:0.5:7 cm), 5 mm base thickness, 5 mm bar spacing, identical ridges for each RF)	Lung, 5 patients, 34 Gy in 1 fx	3 fields	Iterative spot reduction	IMPT	DADR	DRVH, dose rate distributions	Motion management: Robustness: Comments: Plan adaptation: reselecting RF from pre-designed set
**(67) Ramesh2023** doi:10.1002/mp.16009	Hybrid, BP and TB	-	bilateral H&N, 3 patients, single fraction	3 fields BP, 3-4 BP with dose rate optimisation, 9-13 beam TB with DR	Embedded	IMPT, with dose rate objective SDDRO (Gao) /	DADR	DRVH	Robustness: robust scanning method Comments: dose rate objectives,

			(above 50 Gy)	optimisation, 4 BP and 1 TB hybrid		SDDRO-joint (Lin)			robust optimisation
**(27) vandeWater2019** doi:10.1080/0284186x.2019.1627416	TB and BP	*Energy switching*	H&N, 4 patients, 2/6 Gy per fx	5 fields standard (BP), 120 fields in arc arrangement (BP and TB)	Pencil beam resampling	IMPT	DADR	DRVH, Dose rate distributions	Robustness: Comments: Spot reduction necessary
**(68) Kang2022** doi:10.1016/j.ijrobp.2022.01.009	BP and TB	*Universal RS and RC with raytracing* (Universal range shifter with 6 movable plastic plates (1, 2, 3, 7, 7, 14 mm) 3D-printed range compensators)	Lung, 6 patients, 34GyE in 1 fx (SBRT)	5 fields	Spot map optimisation	IMPT	DADR	DRVH	Motion management: averages 4DCT; interplay effect not concerning for single field, but between fields and fractions Robustness: 5 mm setup, 3.5% range
**(69) Wei2021b** doi:10.3390/cancers13225790	BP and TB	*Universal RS and RC with raytracing*	Lung, 10 patients, 34 Gy in 1 fx, 54 Gy in 3 fx (SBRT)	5 fields, equiangular for 34 Gy per fx, 4 fields for 18 Gy per fx (higher dose rates)	Spot map optimisation	IMPT	DADR, ADR (PBS-DR), DTDR (Kang)	DRVH, dose rate distribution	Motion management: Robustness: Comments:
**(29) Krieger2022** doi:10.1002/mp.15459	BP and TB	(downstream energy modulation)	Brain, lung, nasal cavity, pancreas and prostate, 2 patients each, 60 Gy in 30 fx	Single field and multi-fields (7-11 TB, 2-4 BP)	Pencil beam resampling	MCO, IMPT		FLASH effectiveness model, weighted dose	Motion management: Robustness: Comments: only downstream modulated BP considered, no air gap modelled, sensitivity analysis with different fractionation schemes, FLASH

									effect, dose and dose rate threshold
**(25) Schwarz2022** doi:10.1002/mp.15579	BP and TB and LINAC	*3D-RM* (Simeonov et al. (2017) (doi:10.1088/1361-6560/aa87f5)), collapse multi-energy plan (SOBP calculated for 1-8 cm. BP weights converted to quasi-stepless, pyramid-shaped base structure with 1.5x1.5 mm^2 base, pins mounted on modulator)	Lung (TB, 20 GyE in 3fx), Liver metastasis (3D-RM, 25 GyE in 1 fx), Prostate (LINAC, 8 GyE in 5 fx)	3 fields (TB), single field (3D-RM), opposing fields (LINAC)	Pencil beam resampling	MFO (TB), DFO (LINAC)	DADR, sliding window	DR distributions, FLASH coverage	Motion management: ITV from 3 DIBH CTs (lung), single CT (liver) Robustness: Comments: TB intrinsically robust to range uncertainties
**(70) Pennock2023** doi:10.3390/cancers15153828	BP and TB	*Universal RS and RC with raytracing*	Recurrent H&N, 8 patients, 6/10 GyE in 1 fx	Multi field, small air gap, 3 cm RS for H&N	Spot map optimisation	IMPT	DADR	DRVH, dose rate distributions	Motion management: Robustness: 3 mm setup and 3.5% range Comments:
**(71) Wei2022a** doi:10.1002/mp.15894	BP	*Universal RS and RC with raytracing*	Liver, 10 patients, 50 Gy in 5 fx	Clinical arrangements	Spot map optimisation	IMPT	ADR (PBS-DR), DADR, DTDR	V_40Gy/s for ADR, DADR and DTDR, DRVH, DR distributions	Motion management: ITV from averaged 4DCT Robustness: 5 mm setup and 3.5% range Comments: minimum MU per spot limits flexibility
**(72) Wei2022b** doi:10.1016/j.radonc.2022.08.005	BP	*Universal RS and RC with raytracing*	Lung, 10 patients, 34 Gy in 1 fx (SBRT)	2-4 beams (clinical)	Spot map optimisation	IMPT	ADR (PBS-DR)	DRVH, DR distributions	Motion management: ITV from averaged 4DCT Robustness:

									Comments: sensitivity analysis dose threshold
**(73) Lattery2023** doi:10.3390/cancers15184560	BP	*Universal RS and RC with raytracing*	Breast, 10 patients, 5 fx of 8 Gy	Clinical arrangements	Spot map optimisation	IMPT	ADR (PBS-DR), voxel-wise	DRVH, dose rate distributions	Motion management: Robustness: Comments: 5 to 8 mm spot spacing, dose rate coverage depending on threshold and time in-between beams
**(24) Ma2023b** doi:10.1002/mp.16939	BP	*Pin-RFs*, beam model commissioning (Multiple pyramid-shaped ridge pins on a base, height/width of pins according to weight/depth of BP; 5 mm base thickness, 6 mm pin spacing, 6 mm pin width. Widths rounded to nearest integer)	Lung, 3 patients, 34 Gy in 1fx (SBRT)	3 fields	PBD-based spot reduction	IMPT		FLASH effectiveness model	Motion management: Robustness: 5 mm setup, 3.5% range Comments: 7 mm spot spacing and 6 mm pin
**(74) LiuG2023** doi:10.1016/j.ijrobp.2023.05.012	BP (Arc)	-	Brain (16 Gy in 1 fx), lung (40 Gy in 5 fx), prostate (35 Gy in 5 fx)	Sampled from arc	Embedded in SPArc	„Advanced“ IMPT with SPArc	DADR	DR distribution above 5 Gy threshold	Sequential clinical goals and spot-wise DR optimisation, conformity
**(75) LiuR2023** doi:10.1016/j.ijrobp.2023.01.048	BP	Stepwise *ridge pins*	Lung, 3 patients,	3 beams	Sparse ridge filters	Simultaneous dose, DR	DADR	DR distributions, DRVH,	Motion management:

		(Weight (cross-sectional area of step) and thickness (height) pre-calculated in information file with 0.1 mm step size resolution, Sparse ridge filter generation by removing pins and calculation of dose rate)	10 Gy in 5 fx		based on DR	and LET optimisation		LETd (dose-averaged LET)	Robustness: 5 mm setup and 5% range, Comments: 4 Gy per fraction per field for FLASH, sparse RF with more hot spots

Risk of bias assessment

Table 7: Risk of bias assessment for in-vivo pre-clinical studies

	Selection bias			Performance bias		Detection bias		Attrition bias	Reporting bias	Other
**Study**	**Sequence generation**	**Baseline characteristics**	**Allocation concealment**	**Random Housing**	**Blinding**	**Random outcome assessment**	**Blinding2**	**Incomplete outcome data**	**Selective outcome reporting**	**Other sources**
(20) Cunningham2021	Unclear	Low	Unclear	Unclear	Unclear	Unclear	Unclear	Low	Low	Low
(8) Karsch2022	Unclear	Low	Unclear	Unclear	Unclear	Unclear	Unclear	Low	Low	Low
(13) Loo2017	Unclear	Low	Unclear	Unclear	Unclear	Unclear	Unclear	Low	Low	Low
(11) Montay-Gruel2017	Unclear	Low	Unclear	Unclear	Low	Low	Low	Low	Low	Low
(48) Poulsen2024	Low	Low	Low	Low	Low	Low	Low	Low	Low	Low
(15) Ruan2021	Low	Low	Low	Low	Unclear	Low	Unclear	Low	Low	Low
(47) Saade2023	Low	Low	Unclear	Low	Unclear	Unclear	Unclear	Low	Low	Low
(49) Dai2023	Low	Low	Unclear	Low	Unclear	Unclear	Unclear	Low	Low	Low
(50) Mascia2024	Low	Low	Unclear	Unclear	Low	Unclear	Unclear	Low	Low	Low
(51) Alaghband2023	Low	Low	Unclear	Unclear	Unclear	Unclear	Unclear	Low	Low	Low
(52) Allen2023	Low	Low	Unclear	Unclear	Unclear	Unclear	Unclear	Low	Low	Low
(53) Limoli2023	Low	Low	Unclear	Low	Unclear	Unclear	Unclear	Low	Low	Low
(54) Montay-Gruel2021	Low	Low	Low	Unclear	Low	Low	Low	Low	Low	Low
(3) Böhlen2022*	Low	Low	Unclear	Unclear	Unclear	Unclear	Unclear	Unclear	Low	Low

*Based on individual reports included in the study

Table 8: Risk of bias assessment for treatment planning studies

Study	Selection bias	Data quality	Study design	Comparative methods	Outcome measures	Statistical analysis	Reporting transparency	Conflict of interest
(55) Gao2020	High	Unclear	Low	Low	Low	Unclear	Low	Low
(26) vanMarlen2020	Low	Low	Low	Low	Low	Low	Low	High
(56) vanMarlen2021	Low	Low	Low	Low	Low	Low	Low	High

(57) Kang2021	Low	Low	Low	Low	Low	Low	Low	Low
(58) Wei2021a	Low	Low	Low	Low	Low	Low	Low	Low
(59) Wei2022c	Low	Low	Low	Low	Low	Low	Low	Low
(60) Gao2022	High	Unclear	Low	Low	Low	Unclear	Low	Low
(28) Habraken2022	Low	Low	Low	Low	Low	Low	Low	Low
(61) vanMarlen2022	Low	Low	Low	Low	Low	Low	Low	Unclear
(62) vanMarlen2023	High	Low	Low	Low	Low	Unclear	Low	High
(63) JoseSanto2023	Low	Low	Low	Low	Low	Low	Low	Low
(64) Kneepkens2023	Low	Low	Low	Low	Low	Low	Low	High
(65) Lin2021	High	Unclear	Low	Low	Low	Unclear	Low	Low
(66) Ma2023a	Low	Low	Low	Low	Low	Low	Low	Low
(67) Ramesh2023	High	Unclear	Low	Low	Low	Unclear	Low	Low
(27) vandeWater2019	High	Low	Low	Low	Low	Low	Low	Low
(68) Kang2022	Low	Low	Low	Low	Low	Low	Low	Low
(69) Wei2021b	Low	Low	Low	Low	Low	Low	Low	Low
(29) Krieger2022	High	Low	Low	Low	Low	Unclear	Low	High
(25) Schwarz2022	High	Unclear	Low	Low	Low	Unclear	Low	High
(70) Pennock2023	Low	Low	Low	Low	Low	Low	Low	Low
(71) Wei2022a	Low	Low	Low	Low	Low	Low	Low	Low
(72) Wei2022b	Low	Low	Low	Low	Low	Low	Low	Low
(73) Lattery2023	Low	Low	Low	Low	Low	Low	Low	Low
(24) Ma2023b	High	Low	Low	Low	Low	Unclear	Low	Low
(74) LiuG2023	High	Unclear	Low	Low	Low	Unclear	Low	Low
(75) LiuR2023	High	Unclear	Low	Low	Low	Unclear	Low	Low

Selection bias: Were the data selected without bias?
Data Quality: Are the data sources reliable and validated?
Study design: Is the study design appropriate and free from bias?
Comparative measures: Are the methods used for coparison appropriate and consistently applied?
Outcome measures: Are the outcome measures relevant and reliably measured?
Statistical analysis: Are the statistcial methods appropriate and free from bias?
Reporting transparency: Is the study reporting transparent and complete?
Conflict of interest: Are there any potential conflicts of interest?

Impact assessment – scoring details

Table 9: Detailed scoring from author 1

	Conditions and Aspects																				
	FLASH Dose			FLASH Fractionation			FLASH Beam pauses			FLASH Dose rate			FLASH Delivery time			FLASH Radiobiology and Models			FLASH Treatment Mode		
**Treatment Process Steps**	Criticality	Knowledge	Mitigation	Criticality	Knowledge	Mitigation	Criticality	Knowledge	Mitigation	Criticality	Knowledge	Mitigation	Criticality	Knowledge	Mitigation	Criticality	Knowledge	Mitigation	Criticality	Knowledge	Mitigation
Patient Selection	3	2	2	2	2	2	2	2	2							3	2	3	3	2	2
Target Volume Delineation and OAR Contouring							1	1	1				1	1	1						
Treatment Planning System							3	2	2	3	2	1	3	2	2				3	1	1
Dose Prescription and Reporting	3	2	1	3	2	1	3	2	1	3	1	1	2	2	1	2	2	2			
Definition of Dose Objectives	3	2	1	3	2	1	3	2	1	3	1	1	2	2	1	3	2	2			
Choice of Treatment Mode and Beam Directions	3	2	1	2	2	1	3	2	2										3	1	2
Placement of Spots										3	2	1	3	2	1						
Optimisation of Spot Weights				2	2	2	2	2	2	3	2	2							2	2	1
Robustness	3	2	2	2	2	1	2	2	2	2	2	2	3	2	2	2	2	1	2	2	2
Plan Evaluation							2	3	2	2	3	2	2	3	2	3	2	2			
QA and Delivery	2	1	2				3	1	1	3	2	2	3	1	1				3	2	2
	46			36			76			62			54			54			45		

Table 10: Detailed scoring from author 2

	Conditions and Aspects																				
	FLASH Dose			FLASH Fractionation			FLASH Beam pauses			*FLASH Dose rate*			FLASH Delivery time			FLASH Radiobiology and Models			FLASH Treatment Mode		
**Treatment Process Steps**	Criticality	Knowledge	Mitigation	Criticality	Knowledge	Mitigation	Criticality	Knowledge	Mitigation	Criticality	Knowledge	Mitigation	Criticality	Knowledge	Mitigation	Criticality	Knowledge	Mitigation	Criticality	Knowledge	Mitigation
Patient Selection	3	1	2	2	2	2	2	2	2							3	2	3	2	1	2
Target Volume Delineation and OAR Contouring							2	2	2				2	3	2						
Treatment Planning System							3	2	1	3	1	1	3	2	1				2	1	1
Dose Prescription and Reporting	3	1	2	2	2	2	2	2	2	3	1	1	2	2	1	3	2	1			
Definition of Dose Objectives	3	2	2	2	2	2	2	2	2	3	1	1	2	2	1	3	2	1			
Choice of Treatment Mode and Beam Directions	3	1	2	2	2	1	3	2	2										2	1	2
Placement of Spots										3	1	1	3	1	1						
Optimisation of Spot Weights				2	2	2	2	2	2	3	2	2							3	1	1
Robustness	2	2	2	2	1	1	3	3	3	3	2	2	2	2	2	3	2	1	2	2	2
Plan Evaluation							2	3	2	3	2	2	2	3	2	3	2	1			
QA and Delivery	3	1	2				3	1	1	3	2	2	2	1	2				2	2	2
	44			38			100			60			53			42			29		

Table 11: Detailed scoring after consensus from both authors

	**Conditions and Aspects**																				
	FLASH Dose			FLASH Fractionation			FLASH Beam pauses			*FLASH Dose rate*			FLASH Delivery time			FLASH Radiobiology and Models			FLASH Treatment Mode		
**Treatment Process Steps**	Criticality	Knowledge	Mitigation	Criticality	Knowledge	Mitigation	Criticality	Knowledge	Mitigation	Criticality	Knowledge	Mitigation	Criticality	Knowledge	Mitigation	Criticality	Knowledge	Mitigation	Criticality	Knowledge	Mitigation
Patient Selection	3	***2***	2	2	2	2	2	2	2							3	2	3	**2**	**1**	2
Target Volume Delineation and OAR Contouring																					
Treatment Planning System							3	2	**1**	3	***2***	1	3	2	**1**				**2**	1	1
Dose Prescription and Reporting	3	***2***	***2***	**2**	2	**1**	**2**	2	**1**	3	1	1	2	2	1	2	2	**1**			
Definition of Dose Objectives	3	2	***2***	***3***	2	**1**	**2**	2	**1**	3	1	1	2	2	1	3	2	**1**			
Choice of Treatment Mode and Beam Directions	3	***2***	**1**	2	2	1	3	2	2										***3***	1	2
Placement of Spots										3	***2***	1	3	**1**	1						
Optimisation of Spot Weights				2	2	2	2	2	2	3	2	2							**2**	***2***	1
Robustness	**2**	2	2	2	**1**	1	***3***	***3***	***3***	***3***	2	2	***3***	2	2	***3***	2	1	2	2	2
Plan Evaluation							2	3	2	***3***	***3***	2	2	3	2	3	2	**1**			
QA and Delivery	***3***	1	**1**				3	1	1	3	2	2	***3***	1	**1**				**2**	2	2
	53			32			84			72			44			40			32		

towards higher score
towards lower score
